# The impact of skinfolds measurement on somatotype determination in Heath-Carter method

**DOI:** 10.1371/journal.pone.0222100

**Published:** 2019-09-06

**Authors:** Anna Pastuszak, Jan Gajewski, Krzysztof Buśko

**Affiliations:** 1 Team Projects, Institute of Sport—National Research Institute, Warsaw, Poland; 2 Department of Statistics and Information Technology, Józef Piłsudski University of Physical Education, Warsaw, Poland; 3 Department of Anatomy and Biomechanics, Kazimierz Wielki University, Bydgoszcz, Poland; National Taipei University of Nursing and Health Sciences, TAIWAN

## Abstract

**Objectives:**

The study aim was to determine if a difference exists in skinfold thickness measured by two interchangeable approaches; (1) supraspinale skinfold recommended in the Heath-Carter method and (2) iliac crest skinfold measurement. The question arises as to whether each approach has a similar or different effect on endomorphy determination, and whether there is a possibility to estimate the supraspinale skinfold based on other skinfold measurements.

**Methods:**

A group of 186 male and 161 female students participated in this study. Anthropometric examination included all somatic measurements, as recommended in the Heath-Carter protocol, and the iliac crest skinfold measurement. Estimation of the supraspinale skinfold was performed based on the multiple linear regression procedure.

**Results:**

Skinfold thickness measured in the supraspinale and iliac crest differed (p<0.001) in both men (5.41±1.65 mm and 9.55±4.05 mm, respectively) and women (8.87±4.08 mm and 15.20±6.85 mm), respectively. Endomorphy was significantly higher (0.46 in men, 0.63 in women) when the iliac crest skinfold was used. Subscapular skinfold and iliac crest skinfolds were included in the linear regression model for supraspinale skinfold estimation (R^2^ = 0.724, SE = 0.9 mm and R^2^ = 0.947, SE = 2.3 mm for men and women, respectively).

**Conclusion:**

Two common skinfold approaches produced different measurements between the supraspinale and iliac crest skinfolds, which subsequently affected estimated endomorphy. Regression equations for supraspinale skinfold enabled correction of endomorphy in the case of improperly applied measurement (i.e. iliac crest) and thus, could allow for uniform somatotype estimation according to Carter and Heath approach.

## Introduction

The Heath-Carter anthropometric method,[1–2] is one of the commonly used approaches for somatotyping in anthropology, sport and health science. This methodology has been employed to document body types in elite athletes from various sports, including gymnastics,[3] climbing,[4–6] alpine skiing,[7], football,[8–9], handball,[10–11] water polo,[12–13] and combat sports,[14–20]. The contribution of individual body components, as somatic effects of sports training has also been examined,[21]. Comparison of somatotypes revealed different body types in athletes compared to non-athletes,[22–26], especially in terms of the contribution of endomorphy and mesomorphy. Studies in the social sciences have additional highlighted a number of somatotypes in populations with some dependency on social status,[27] and ethnic, cultural and geographical factors,[28–30]. This method to estimate individual somatotype of patients has also been used to identify predictors of illness,[31–36].

The researchers who have analysed somatotype components, in terms of the above-mentioned factors, have mainly compared their observations with the results obtained by other authors or analysed variability of body type over time. In recent years, a comparison of published reports using the Heath-Carter method revealed that, in certain cases, reasoning may have led to erroneous conclusions due to different measurement techniques.

Mistakes made during somatotype estimation primarily arise from the measurement above the iliac skinfold. The supraspinale skinfold method defines this skinfold as a diagonal fold raised five to seven centimetres above the anterior superior iliac spine on a line to the anterior axillary border and on a diagonal line going downwards and inwards at 45°,[1–2, 37]. Many researchers,[20, 27, 34–35, 38–39] have measured the iliac crest skinfold as a diagonal fold raised immediately above the crest of the ilium on a vertical line from the mid-axilla,[37]. Measured in the same way, the suprailiac skinfold was referred to body fat evaluated by Durnin and Womersley,[40]. In addition, the same name (suprailiac skinfold) was used in early publications of the Heath-Carter method,[41–43], but in subsequent papers the supraspinale skinfold approach was applied,[1–2, 37]. This change in nomenclature led some researchers,[44, 5, 36, 45, 46, 28] to use the same skinfold measurement for evaluating of both somatotype and body tissue composition using the Durnin and Womersley method,[40]. This confusion may lead to false conclusions being drawn by those studies employing the incorrect skinfold measurement and limits effective comparisons between studies in this area.

Given the aforementioned issues, the aim of this study was to determine whether a difference exists in measured skinfold thickness between the supraspinale skinfold, as recommended in the Heath-Carter method, and the iliac crest skinfold measurement, which have been used interchangeably. The question arises whether the measurements in these two different body locations influence endomorphy during somatotype determination, and whether there is a possibility to estimate the supraspinale skinfold based on the measurements defined in the Carter and Heath method,[1–2].

## Methods

### Ethics statement

This study was approved by the Senate Ethics Committee of the Josef Pilsudski University of Physical Education in Warsaw, Poland (SKE 001-82-1/2010). The study procedures were implemented according to the Declaration of Helsinki. All participants gave their written consent after receiving information on the study purpose, test procedures and benefits. Furthermore, they were made aware of the possibility to withdraw their consent at any time for any reason.

### Participants

A cohort of 186 male and 161 female second-year students from the Faculty of Physical Education, the University of Physical Education, Warsaw, volunteered for this study. Testing was conducted between the months of October and November in 2012, 2013, and 2014. The physical characteristics of participants are shown in Table 1.

**Table 1:** Anthropometric characteristics of students from the University of Physical Education. Data are expressed as means and standard deviations (SD).

	Male students (N = 186)	Female students (N = 161)
Age (years)	20.7±1.4	20.8±0.9
Height (cm)	179.1±6.4	163.7±6.9
Body mass (kg)	74.4±7.5	57.4±7.6
Body mass index (kg/m^2^)	23.2±1.7	21.4±2.3

### Anthropology measurements

All participants attended an anthropometric examination in a biomechanics laboratory. The following variables were collected: body height and body mass, five skinfolds (triceps, subscapular, iliac crest, supraspinale, medial calf). All measurements were taken by the same researcher according to the International Standards for Anthropometric Assessment,[37] and the Heath-Carter methodology,[1, 2]. Body height was measured using a SiberHegner anthropometer (Switzerland), body mass using electronic scales (Tanita TBF 300, Japan) and skinfolds using a Harpenden skinfold calliper. Individual height-corrected endomorphy was subsequently estimated by a validated formula,[1, 2] as follows:

$$Endomorphy=‐0.7182+0.1451\;(\sum )‐0.00068\;(\sum^{2})+0.0000014\;(\sum^{3})$$

where: Σ = (sum of triceps, subscapular and supraspinale skinfolds) multiplied by (170.18/height in cm).

### Statistical analyses

Data normality was tested using the Shapiro-Wilk test and diagnostic plots. Since the data distributions were right-skewed, the nonparametric Wilcoxon test was used for comparison of supraspinale, iliac crest skinfold and endomorphy scores. Effect size statistics were calculated, as $r=\frac{Z}{\sqrt{2n}}$ and can be interpreted using Cohen’s,[47] criteria; 0.1 = small effect, 0.3 = medium effect, 0.5 = large effect. Estimation of the supraspinale skinfold was performed using multiple linear regression with a backward stepwise procedure. The explanatory variables included skinfold measurements (triceps, subscapular, iliac crest, medial calf), body height and body mass. The regression procedure was applied using both linear and logarithmic models. Statistical significance was set at an alpha level of p<0.05. Descriptive statistics include means and standard deviations (SD) for all variables. All statistical analyses were performed using Statistica (data analysis software system), version 13, (TIBCO Software Inc., 2017).

## Results

The measurements for males and females are depicted in Tables 2 and 3, respectively.

**Table 2:** Skinfold thickness and endomorphy in male students from the University of Physical Education. Data are expressed as means and standard deviations (SD).

Male students (N = 186)	Supraspinale skinfold	Iliac crest skinfold	Z	p	Effect size r
Skinfold thickness (mm)	5.41±1.65	9.55±4.05*	11.82	<0.001	0.613
Sum of skinfolds used to calculate endomorphy (mm)	20.1±4.76	24.1±6.68*	11.82	<0.001	0.613
Height-corrected endomorphy	1.92±0.54	2.38±0.74*	11.82	<0.001	0.613

Note

*–means differ significantly compared to the supraspinale skinfold; p<0.001.

**Table 3:** Skinfold thickness and endomorphy in female students from the University of Physical Education. Data are expressed as means and standard deviations (SD).

Female students (N = 161)	Supraspinale skinfold	Iliac crest skinfold	Z	p	Effect size r
Skinfold thickness (mm)	8.87±4.08	15.20±6.85*	10.73	<0.001	0.596
Sum of skinfolds used to calculate endomorphy (mm)	35.5±10.9	42.1±16.61*	10.73	<0.001	0.596
Height-corrected endomorphy	3.57±1.05	4.20±1.22*	10.76	<0.001	0.600

Note

*–means differ significantly compared to the supraspinale skinfold; p<0.001.

Skinfold thickness at the two locations (supraspinale and iliac crest) were found to be significantly different in both men (p<0.001, effect size r = 0.613) and women (p<0.001, r = 0.596). The mean thickness of the supraspinale skinfold was around 35% less than that measured at the iliac crest (4.14 mm and 6.33 mm, in terms of gender, respectively). These differences in skinfold thickness also had significant effect on the sum of the three skinfolds defined during calculation of endomorphy, which changed the point score for both men (p<0.001, effect size r = 0.613), and women (p<0.001, effect size r = 0.600). Notably, endomorphy decreased when the supraspinale measurement was considered, or it increased when calculated using the iliac crest skinfold about half a point (0.46 in men, 0.63 in women).

**Fig 1:**
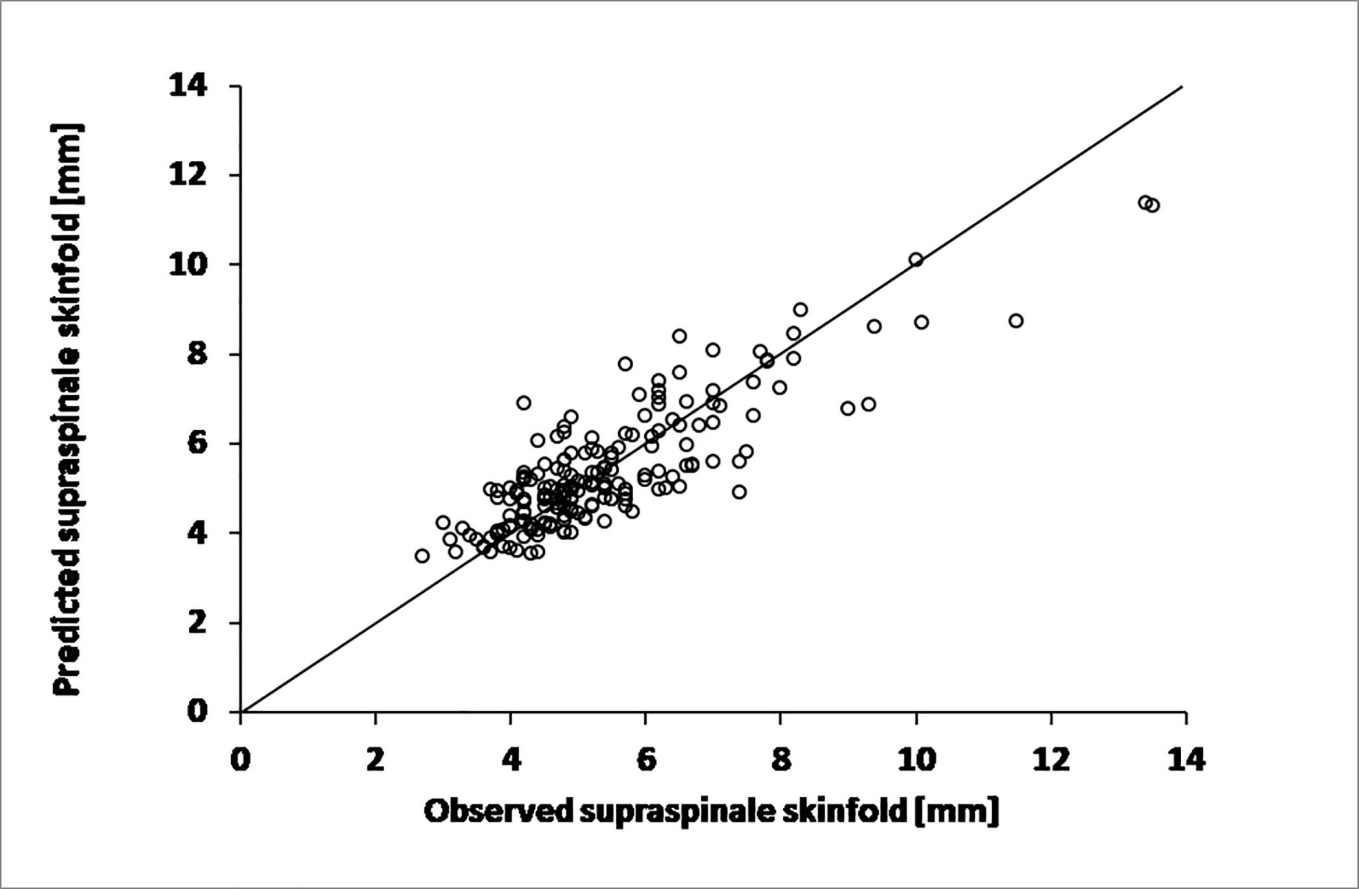
Predicted vs. observed values of supraspinale skinfold thickness obtained using the multiple regression model based on thicknesses of subscapular and iliac crest skinfolds for male subjects.

**Fig 2:**
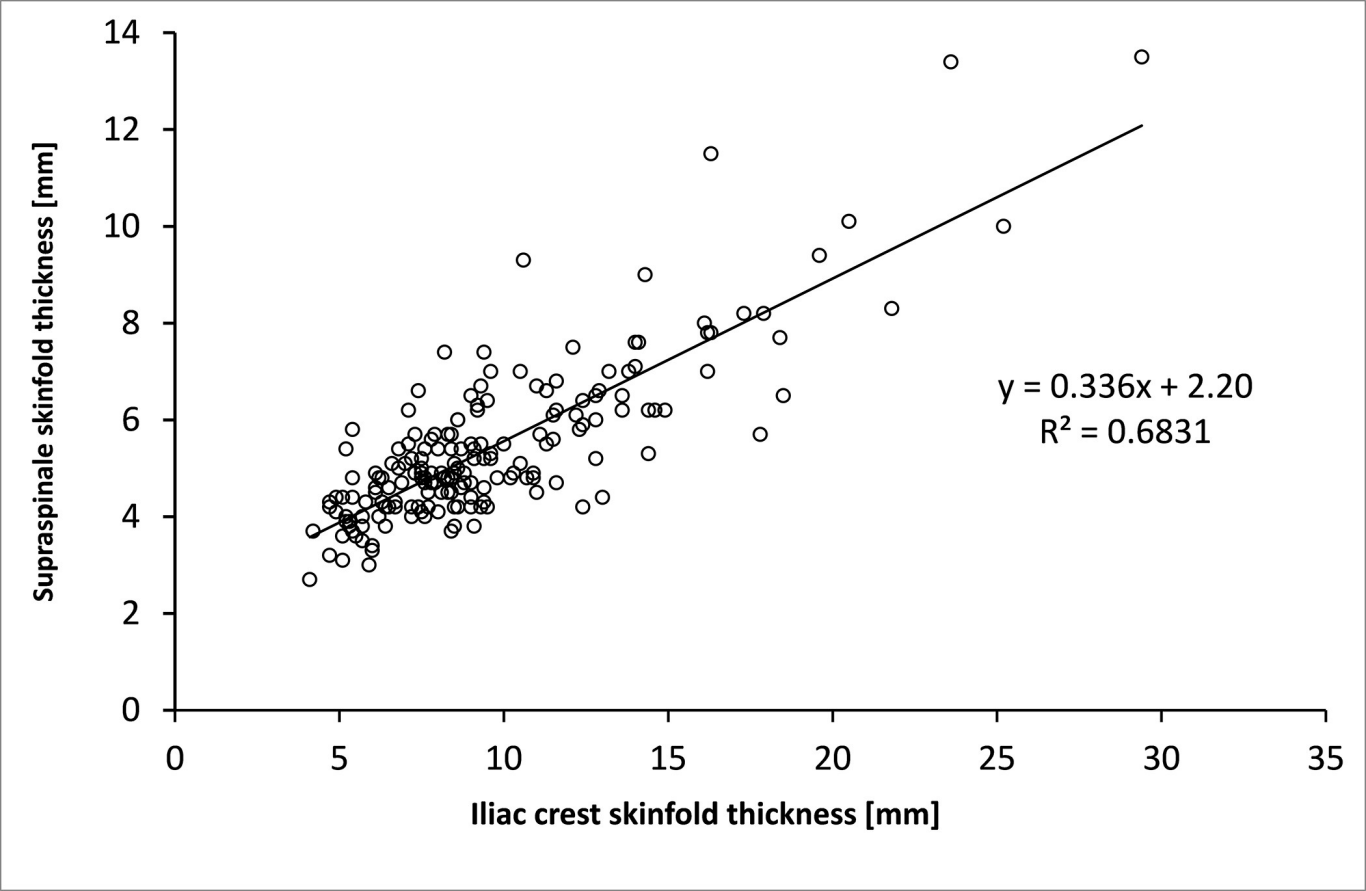
Supraspinale skinfold thickness as a linear function of iliac crest skinfold thickness in male subjects.

**Fig 3:**
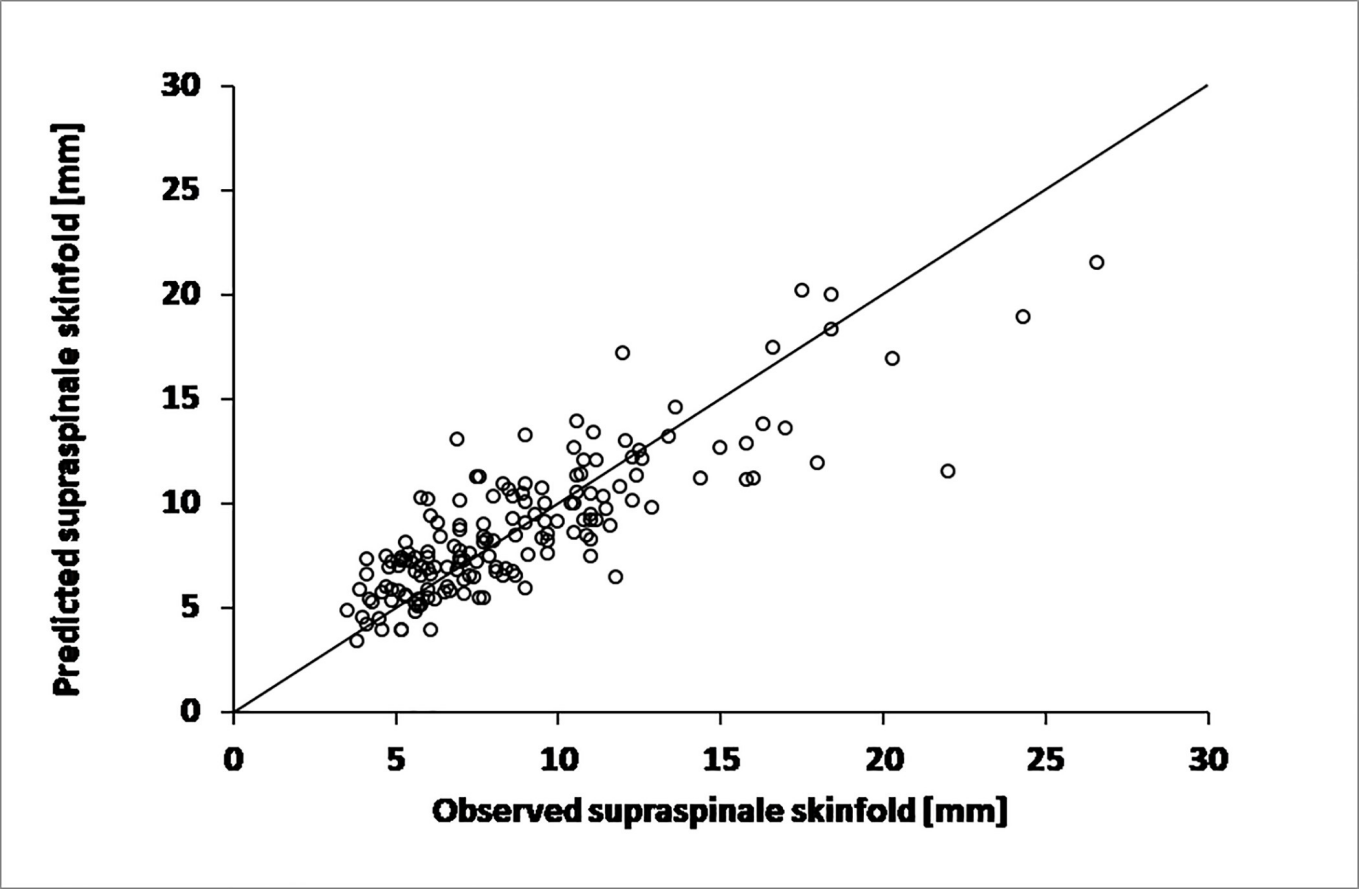
Predicted vs. observed values of supraspinale skinfold thickness obtained using the multiple regression model based on thicknesses of subscapular and iliac crest skinfolds for female subjects.

The regression models are shown in Figs 1, 2 and 3. Only two variables (subscapular skinfold and iliac crest skinfolds) were included in the final model (for women and men, respectively) following the stepwise regression procedure. For men (Fig 1), the following equation was obtained: supraspinale skinfold (mm) = 0.1864·(subscapular skinfold mm) + 0.2750·(iliac crest skinfold mm) + 1.0 with a strong model fit (R^2^ = 0.724, SE = 0.9 mm).

As seen in Fig 2, the estimation with similar error can also be made only based on the iliac crest skinfold (mm): supraspinale skinfold (mm) = 0.3360·(iliac crest skinfold mm) + 2.2 with model fit of R^2^ = 0.683, SE = 1.0 mm.

For women (Fig 3), the formula adopted the following form (model without the constant).: supraspinale skinfold (mm) = 0.4242·(subscapular skinfold mm) + 0.2534·(iliac crest skinfold mm) with a very strong fit (R^2^ = 0.947; SE = 2.3 mm).

The regression procedures were repeated using a logarithmic model. However, greater errors were obtained for the standard estimations. They were 1.6 mm and 2.5 mm for men and women, respectively. Therefore, the decision was made to use linear relationships.

## Discussion

The Heath-Carter method,[1–2] represents a quantitative method for evaluating somatotype in both athletic and non-athletic populations and itself is a modification of an earlier somatoscopic method proposed by Sheldon,[48]. The first attempt to modify the Sheldon method was made by Parnell,[41] who introduced measurements to objectify this evaluation process, subsequent changes in somatotype methodology introduced by Honeyman-Heat,[42–43]. In 1990,[1], the creators of the Heath-Carter method considered these modifications and presented a standardized methodology for somatotype assessment,[1–2].

From the literature review on somatotype evaluated by Heath—Carter method results, that the researchers using different technics of measurements. Some of them measured suprailiac skinfold,[24, 29, 45] consistent with the measurement of iliac crest skinfold,[49]. Usually, most authors have used the incorrect name suprailiac skinfold instead of supraspinale skinfold,[8, 10–11, 16, 22, 25–26, 32, 50], despite their referring to the International Standards for Anthropometric Assessment protocol,[37] and the Heath-Carter methodology,[1], which introduced the new nomenclature. Other group of researchers who presented somatotypes of athletes measured abdominal skinfold,[23,51] skinfold completely different than that indicated in the measurement technique specified in the somatotype methodology. Furthermore some authors failed to provide information about measurements performed in their studies,[4, 13, 24]. It seems that such errors are generated in papers published in journals with high impact factors,[4–5, 8, 10–11, 13, 20, 22–23, 26–28, 32–33, 39, 51] as well as “less prestigious” ones,[16, 24–25, 34, 36, 38, 44–46, 50].

The greatest confusion connected with using measurements in body locations other than the supraspinale to evaluate somatotype is associated with nomenclature. Previous suprailiac skinfold, defined in the Heath-Carter methodology,[42–43] was measured in a different place above the iliac crest, then suprailiac in the method evaluate the level of total body fat developed by Durnin and Womersley,[40]. This mistake committed the designers of the popular software for electronic estimation of total body fat, who used equations developed by the method's originators,[40] defined the measurement in other body locations,[52].

The aim of the present study was to examine whether supraspinale and iliac crest skinfold thickness, which are both commonly termed the suprailiac skinfold, measured in different body locations differ from each other. Our findings demonstrated that the difference is statistically significant with a large effect size (r = 0.613 for men and r = 0.596 for women). The mean thickness of the iliac crest skinfold was nearly twice as big as the supraspinale skinfold (9.55±4.05 and 5.41±1.65, respectively) in physically active university students from Warsaw). The measurement results showed consistency with the results obtained in groups of amateur Spanish soccer players (12.7 ± 5.1 and 7.0 ± 3.3),[9] and college athletes who participated in the Spanish university triathlon championships (11.78 ± 4.14 and 7.42 ± 2.53),[53]. Furthermore, the highest values of the measurement of the iliac crest skinfolds then supraspinale were found for the substantially older groups of Spanish water polo players (16.6±8.0 and 9.8±4.3, respectively),[12] and karate athletes (16.2±8.2 and 10.8±7.2, respectively),[17]. In young handball players, a decline was documented in the value of iliac crest and supraspinale skinfolds in age categories from 10 to 14 years (from 13.5 to 12.5 and 7.5 to 6.5, respectively). All the above presented data, published by other authors, point the difference between the compared skinfolds, measured in different body locations. In our group of female university students from Warsaw, the results of the compared measurements supraspinale skinfold (8.87±4.08) and iliac crest (15.20±6.85), were similar to those found in the female participants of the Pilates Mat Program following a 16-week training programme (12.37 ± 4.16 and 16.00 ± 4.00, respectively),[21]. Substantially lower values of the supraspinale and iliac crest skinfolds were documented in a group of Spanish female gymnasts (4.74–6.05 and 6.68–8.56, respectively, depending on the category). It is remarkable that there is a difference in thickness of the compared skinfolds even in very slim female athletes,[3]. Detailed measurements which took into consideration the supraspinale and iliac crest skinfolds were also taken in overweight, premenopausal Uruguayan women to analyse detailed anthropometric characteristics for risk of breast cancer,[35]. The values of one of the compared skinfold measurements were over twice as high as the other in both women from the risk group (22.56 ± 11.10 and 53.74 ± 20.25) and those from the control group (19.91± 9.23 and 48.49± 18.73). The results of the measurements of the supraspinale and iliac crest skinfolds in obtained by other authors confirm the nearly two-fold difference in the thickness between the skinfolds in both men and women, so indicated in our study. Such difference, also had an effect on the value of the estimated endomorphy (p<0.001). This is likely to have caused differences in this somatotype component in systematic reviews,[54] that compared results obtained by different authors. One example, is the endomorphy ranging, from 2.1 to 3.2 points in Brazilian jiu-jitsu athletes from the same weight category,[14]. The proposed equations that can be used to predict the supraspinale skinfold allow for correction of the erroneously estimated endomorphy and consequently, allow the methodology for estimation of the somatotype to be made uniform. Otherwise the equations can be a way of correcting data in the case of improperly applied measurement on the iliac crest. However, it should be remembered that the proposed formulas can be properly applied for data correction only for young subjects of similar age as those tested in our study.

### Practical implications

The study findings verify the need for standardizing methodologies,[1,2], vocabulary and measurement techniques, especially those concerning skinfolds,[37] when estimating somatotype from skinfold measurements. The supraspinale skinfold should not be used to evaluate endomorphy by means of the Heath-Carter method. Using skinfolds measured via the iliac crest, the proposed equations that predict the value of the supraspinale skinfold can be used for correction of the erroneously estimated endomorphy.

## Conclusion

The results confirm the difference in thickness between the supraspinale and iliac crest skinfolds, which affects the estimated endomorphy. The equations for supraspinale skinfold prediction allow for correction of the endomorphy in the case of improperly applied measurement on the iliac crest, and consequently allow the methodology for somatotype estimation to be made uniform according to Carter and Heath. The proposed equations are recommended for adult populations of around 20 years old, both sexes without being overweight, such as the ages of examined group.

## Supporting information

S1 Data Data to calculation. (XLS)
